# Patient-facing materials for hip and knee preservation surgery are written well above recommended reading level

**DOI:** 10.1093/jhps/hnaf066

**Published:** 2025-11-04

**Authors:** Brendan Gleason, Clark Chen, Keenan Sobol, Julien Aoyama, Gregory Schneider, S Ali Ghasemi

**Affiliations:** Jefferson Einstein Philadelphia Hospital, Department of Orthopaedic Surgery, 5501 Old York Rd, Philadelphia, PA 19141, United States; Jefferson Einstein Philadelphia Hospital, Department of Orthopaedic Surgery, 5501 Old York Rd, Philadelphia, PA 19141, United States; Jefferson Einstein Philadelphia Hospital, Department of Orthopaedic Surgery, 5501 Old York Rd, Philadelphia, PA 19141, United States; Jefferson Einstein Philadelphia Hospital, Department of Orthopaedic Surgery, 5501 Old York Rd, Philadelphia, PA 19141, United States; Jefferson Einstein Philadelphia Hospital, Department of Orthopaedic Surgery, 5501 Old York Rd, Philadelphia, PA 19141, United States; Jefferson Einstein Philadelphia Hospital, Department of Orthopaedic Surgery, 5501 Old York Rd, Philadelphia, PA 19141, United States

**Keywords:** joint preservation, hip, knee, education, readability

## Abstract

Hip and knee preservation have rapidly expanded over the past decade. As technology and surgeon familiarity have increased, patient interest in this field has also grown. Health literacy data demonstrates that up to 43% of US adults have basic or below-basic reading skills. Federal and medical agencies recommend patient facing materials (PFMs) be written no higher than a sixth to eighth grade level. We identified PFMs pertaining to hip and knee preservation surgery from top orthopaedic institutions, as ranked by the 2025 US News & World Report. PFMs were assessed using validated readability formulas. We then used Spearman rank correlation to examine the association between the hospital’s ranking and the readability of its PFMs. A multivariate analysis was also conducted to explore whether institutional characteristics had any significant relationship with readability scores. PFMs for hip and knee preservation surgery provided by highly ranked orthopaedic institutions were commonly written well above the recommended sixth to eighth grade reading level. There was no significant statistical relationship between institutional characteristics and readability. PFMs for hip and knee preservation surgery are widely available but are written at a much higher reading level than is recommended. To increase accessibility, enhance healthcare literacy, and improve patient outcomes, institutions should simplify these materials to meet recommended readability standards. The gap between the reading level of available materials and the literacy of the target patient population may hinder patient comprehension, engagement, and informed consent, potentially impacting healthcare outcomes.

## INTRODUCTION

Hip and knee preservation techniques including arthroscopy, cartilage restoration, osteotomies, and biologic resurfacing have rapidly expanded over the past decade [1]. These joint saving options are especially important for the growing population of young, athletic patients who wish to avoid or defer arthroplasty. Patient facing materials (PFMs) have been published by many hospitals and orthopaedic practices to help enhance patient understanding of the relatively new and evolving techniques in hip and knee preservation surgery. Health-literacy data show that up to 43% of US adults have basic or below-basic reading skills, with an average eighth-grade reading ability [2]. Improving health literacy is a central focus of Healthy People 2030 [3]. Evaluation of the readability of medical texts and PFMs is becoming increasingly popular and objective measures and calculators of readability have become more widely available to facilitate this evaluation.

The National Institute of Health and the American Medical Association recommend PFMs be written no higher than a sixth-grade level [2]. Similarly, the Center of Disease Control (CDC) allows up to an eighth-grade level [3]. However, orthopaedic patient materials routinely exceed these thresholds [4–6]. While this is far from an isolated orthopaedic problem, clear and accessible PFMs are critical for informed decision-making and optimal outcomes [7–11]. Studies have shown that patient education materials routinely exceed the recommended reading levels [8]. Specifically, a 20 year analysis found that only 2.1% of patient education materials published in medical journals were written at or below a sixth-grade reading level [12]. Assessing the readability of PFMs pertaining to hip and knee preservation surgery can highlight the discrepancy between content complexity and patient literacy thereby supporting efforts to minimize this gap.

## MATERIALS AND METHODS

We identified PFMs pertaining to hip and knee preservation surgery from the top 25 orthopaedic specialty institutions, as ranked by the 2025 US News & World Report [13]. Materials were identified with web search including the institution name and the keywords of hip preservation or knee preservation. Webpages were downloaded and converted into pain text format and assessed using 10 validated readability formulas: Flesch–Kincaid Grade Level, Flesch Reading Ease, Gunning Fog Index, Coleman–Liau Index, Simple Measure of Gobbledygook (SMOG) Index, Automated Readability Index (ARI), Linsear Write Grade Level Formula, FORCAST, and New Dale–Chall Readability, and Average Reading Level Consensus (Tables 1 and 2). Variance inflation factors were calculated to check for multicollinearity among these readability measures.

**Table 1:** Individual reading scores for online hip preservation resources at top orthopaedic institutions.

**Institution rank**	**Average Reading Level (Consensus)**	**Automated Readability Index (ARI)**	**Gunning Fog Index (GFI)**	**Flesch–Kincaid Grade Level**	**Coleman–Liau Index**	**SMOG Index**	**Linsear Write Grade**	**FORCAST**	**New Dale–Chall Readability**	**Flesch Reading Ease**
1	11.4	10.6	12.7	10.3	12.1	10.4	14.4	11.0	25.0	49.0
2	12.0	11.7	13.6	11.2	13.2	10.5	10.4	11.5	23.0	43.0
3	12.2	13.0	13.5	12.2	12.3	11.6	13.3	11.0	23.0	45.0
4	13.7	14.0	16.3	13.4	18.8	9.5	34.7	13.8	8.0	12.0
5	10.6	9.7	13.2	9.3	12.2	9.6	11.6	10.9	27.0	51.0
6	12.0	11.0	13.2	10.8	12.9	10.2	13.3	11.2	21.0	44.0
7	14.1	14.5	18.0	13.3	19.2	9.7	27.7	14.1	7.0	12.0
8	15.4	17.8	17.8	15.9	16.9	15.0	17.0	12.5	9.0	21.0
9	11.5	10.6	12.8	10.7	11.9	10.6	16.6	11.5	23.0	46.0
10	12.2	11.9	14.0	10.5	14.6	10.5	19.3	11.6	21.0	43.0
11	12.1	11.6	12.2	10.7	13.7	10.1	18.7	11.6	20.0	44.0
12	12.2	11.6	13.2	10.9	14.2	10.4	17.3	11.8	20.0	41.0
13	9.9	9.4	11.3	8.8	10.6	9.1	8.5	10.4	31.0	59.0
14	12.0	11.7	13.6	11.2	13.2	10.5	10.4	11.5	23.0	43.0
15	14.5	15.9	16.5	14.6	16.0	13.9	21.0	12.2	11.0	28.0
16	11.5	11.1	13.1	10.0	14.0	9.9	16.5	11.6	23.0	46.0
17	10.7	10.1	12.3	9.5	12.5	9.7	13.5	11.2	25.0	51.0
18	11.1	11.4	12.6	10.7	10.9	10.6	12.1	10.2	27.0	53.0
19	7.9	7.3	9.2	7.1	7.8	7.7	7.4	9.4	37.0	71.0
20	12.0	12.6	12.9	11.5	12.3	11.3	12.7	10.8	23.0	48.0
21	14.2	15.2	16.5	14.0	15.7	12.9	13.8	12.2	15.0	29.0
22	7.4	5.9	8.4	6.0	7.9	6.9	10.2	9.9	38.0	73.0
23	11.3	9.8	13.4	9.9	13.5	9.1	13.3	12.1	23.0	42.0
24	9.3	8.8	10.2	8.4	10.6	8.5	11.2	10.0	30.0	60.0
25	15.1	18.5	16.1	16.6	15.5	14.7	18.9	12.0	10.0	24.0
										
Mean	11.9	11.8	13.5	11.1	13.3	10.5	15.4	11.4	21.7	43.1
SD	2.0	3.0	2.4	2.5	2.8	1.9	6.0	1.1	8.2	15.5

**Table 2:** Individual reading scores for online knee preservation resources at top orthopaedic institutions.

**Institution rank**	**Average Reading Level Consensus**	**Automated Readability Index (ARI)**	**Flesch Reading Ease**	**Gunning Fog Index**	**Flesch–Kincaid Grade Level**	**Coleman–Liau Index**	**Simple Measure of Gobbledygook (SMOG) Index**	**Linsear Write Formula**	**FORCAST**	**New Dale–Chall Readability**
1	12.3	12.0	46.0	13.5	11.8	11.4	11.6	12.5	11.1	19.1
2	12.0	11.4	46.0	12.1	10.9	12.4	10.4	10.7	11.8	19.1
3	13.5	14.1	30.0	14.0	13.2	15.8	11.7	16.3	12.0	14.6
4	11.6	10.4	45.0	13.5	10.6	12.5	10.4	14.2	11.2	25.9
5	14.8	15.4	16.0	17.1	14.2	19.3	12.0	22.0	13.9	9.7
6	12.6	13.6	45.0	12.8	12.0	13.3	11.1	12.7	11.3	20.3
7	12.1	12.3	48.0	14.4	11.3	12.1	11.3	12.4	10.6	22.8
8	12.2	11.2	45.0	13.9	11.0	12.2	11.1	10.1	11.2	19.7
9	12.4	12.5	42.0	11.7	10.9	14.9	10.4	20.1	12.2	20.2
10	9.5	8.3	62.0	11.6	7.9	9.9	8.7	6.8	10.1	26.1
11	12.6	11.7	45.0	15.1	10.8	13.1	11.4	10.6	11.1	11.7
12	13.8	14.3	16.0	17.1	13.1	19.1	9.9	17.2	13.6	4.4
13	10.6	11.1	52.0	11.6	9.9	12.5	9.8	10.3	10.9	28.8
14	12.8	13.0	40.0	13.6	11.9	14.3	11.7	21.8	11.6	19.2
15	11.6	11.2	42.0	13.8	10.0	14.8	9.6	16.8	11.8	24.3
16	12.0	12.1	49.0	14.2	11.2	11.9	11.3	12.5	10.5	23.2
17	12.7	12.7	38.0	13.3	12.1	13.8	11.4	16.4	11.8	17.2
18	12.5	11.7	38.0	13.6	11.1	14.5	10.3	13.0	12.2	17.7
19	13.3	14.3	38.0	14.9	12.9	14.3	12.6	14.0	11.3	21.0
20	7.3	5.4	74.0	9.2	5.6	7.5	7.3	6.9	9.6	37.0
21	10.3	10.0	54.0	10.8	8.7	12.6	8.8	15.5	11.1	26.4
22	12.5	12.2	44.0	14.2	11.6	12.8	11.5	15.3	10.9	20.0
23	13.2	15.4	42.0	15.0	13.9	12.3	12.7	15.9	10.4	17.0
24	8.9	7.8	62.0	9.9	8.3	9.0	8.2	12.3	10.2	34.0
25	6.1	4.9	79.0	7.5	4.8	7.1	6.0	6.8	9.5	40.0
										
Average	11.7	11.6	45.5	13.1	10.8	12.9	10.4	13.7	11.3	21.6
Standard deviation	2.0	2.7	14.2	2.2	2.3	2.9	1.6	4.2	1.1	8.0

Average Reading Level Consensus utilizes eight of the previous readability formulas to produce a grade level of specific text and was used to determine whether the PFMs available from each institution met sixth or eighth grade reading level targets.

We then used Spearman rank correlation to examine the association between the hospital’s ranking and the readability of its PFMs. A multivariate analysis was also conducted to explore whether institutional characteristics geographic region, public versus private status or the inclusion of multimedia content, videos, and infographics had any significant relationship with readability scores. A total of 25 hip preservation surgery webpages and 25 knee preservation surgery webpages from each of the top-ranked orthopaedic hospitals were analysed for readability.

## RESULTS

A total of 25 hip preservation surgery webpages from each of the top-ranked orthopaedic hospitals were analysed for readability. None met the NIH’s sixth-grade target, and only two pages (8%) achieved the CDC’s eighth-grade threshold according to the Average Reading Level Consensus (Fig. 1). The vast majority (92%) exceeded an eighth-grade level. Across all materials, the mean Flesch–Kincaid Grade Level (FKGL) was 11.7 ± 2.0, ranging from approximately 8.5 (eighth grade) to 15.0 (college sophomore). The average Flesch Reading Ease score was 50 ± 10, indicating ‘fairly difficult’ text (scores <60). Other indices including the Gunning Fog (mean: 13; SD: 2.4), SMOG (mean: 11; SD: 1.93), Coleman–Liau (mean:13; SD: 2.8), ARI (mean: 12; SD: 3.0), Linsear Write (mean: 15; SD: 6.0), FORCAST (mean: 11; SD: 1.1), and New Dale–Chall (mean: 21.7; SD: 8.2), all similarly reflected high school to college-level complexity (Fig. 2). Multimedia videos were present in 15 webpages. Spearman rank correlation between US News ranking and FKGL yielded ρ = −0.10 (*P* = .64), indicating no significant association between institutional rank and readability. Readability metrics were highly collinear (pairwise Pearson *r* > 0.8), confirming that regardless of formula, these materials uniformly exceeded recommended reading levels for the general adult population.

**Figure 1:**
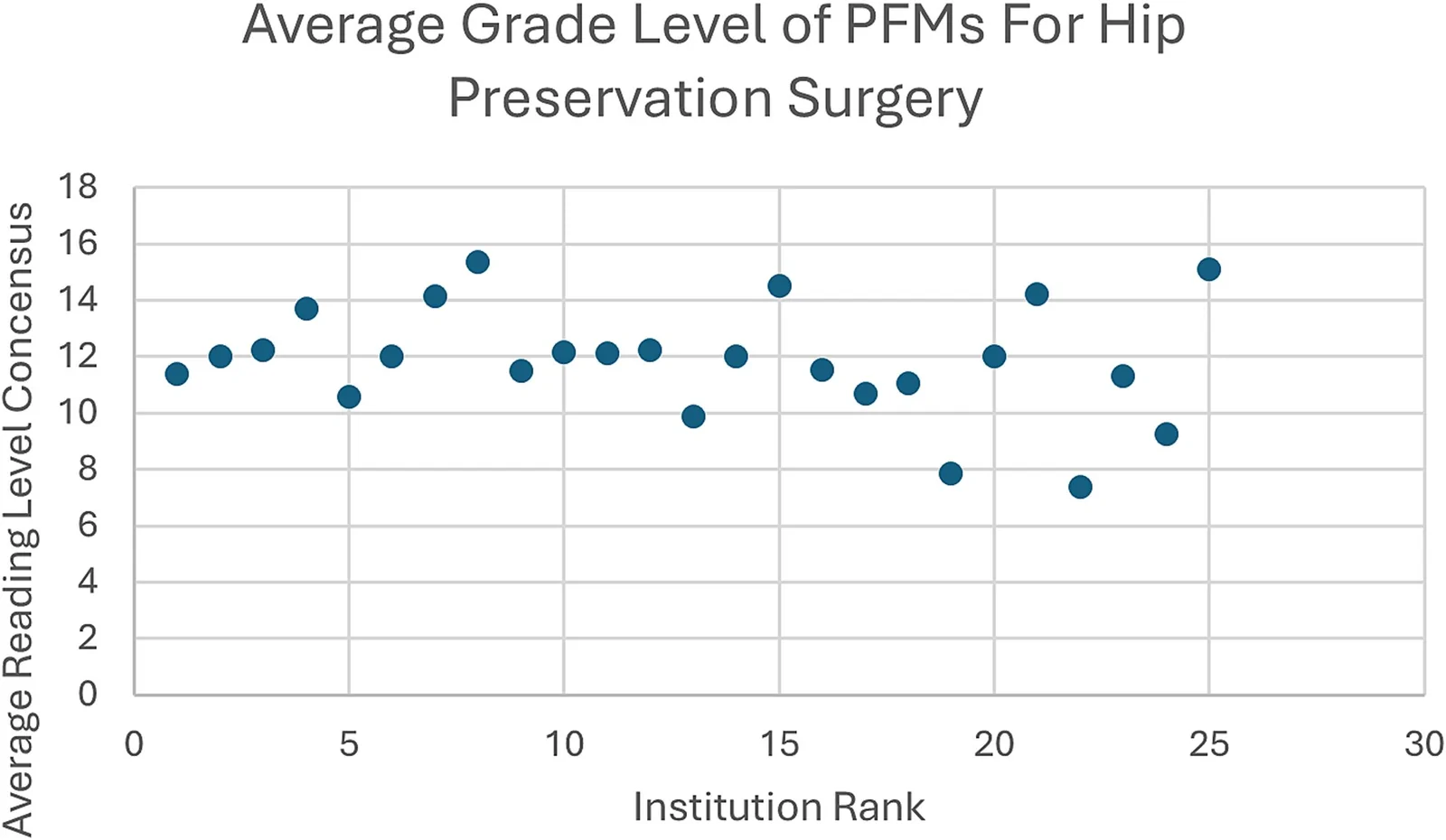
Average grade level of online PFMs for hip preservation surgery provided by top US orthopaedic institutions as graded by the Average Reading Level Consensus. This metric combines 8 validated readability formulas to provide an average reading level of a single text or document.

A total of 25 knee preservation surgery webpages from each of the top ranked orthopaedic hospitals were analysed for readability. One page (4%) approached the NIH’s sixth-grade reading-level target with Average Reading Level Consensus of 6.12, and only two pages (8%) achieved the CDC’s eighth-grade cutoff (Fig. 3). Across all materials, the mean FKGL was 10.8 (SD:2.3), The average Flesch Reading Ease score was 46 (SD: 14.2). Other readability indices showed similar results, uniformly suggesting high school to college-level complexity. The Gunning Fog Index averaged 13.1 (SD 2.2), the SMOG Index 10.4 (SD 1.6), the Coleman–Liau Index 12.9 (SD 2.9), the Automated Readability Index 11.6 (SD 2.7), the Linsear Write Formula 13.7 (SD 4.2), the FORCAST formula 11.3 (SD 1.1), and the New Dale–Chall score 21.6 (SD 8.0) (Fig. 4). Multimedia videos were present in 5 of the 25 webpages (20%). There was no significant relationship between a hospital’s national rank and the readability of its webpage. The Spearman’s rank correlation between US News rank between rank and Flesch Reading Ease was ρ = 0.22 (*P* = .29), indicating no meaningful association. All the readability metrics were highly collinear (Pearson correlation *r* > 0.8), confirming that these patient materials uniformly exceeded recommended reading levels for the general adult population. Finally, a multivariate regression analysis (considering hospital geographic region, public versus private status, and the presence of multimedia content on the page) found no significant predictors of readability (*P* = .12), suggesting that these institutional factors did not substantially influence the reading level of the knee preservation webpages.

**Figure 2:**
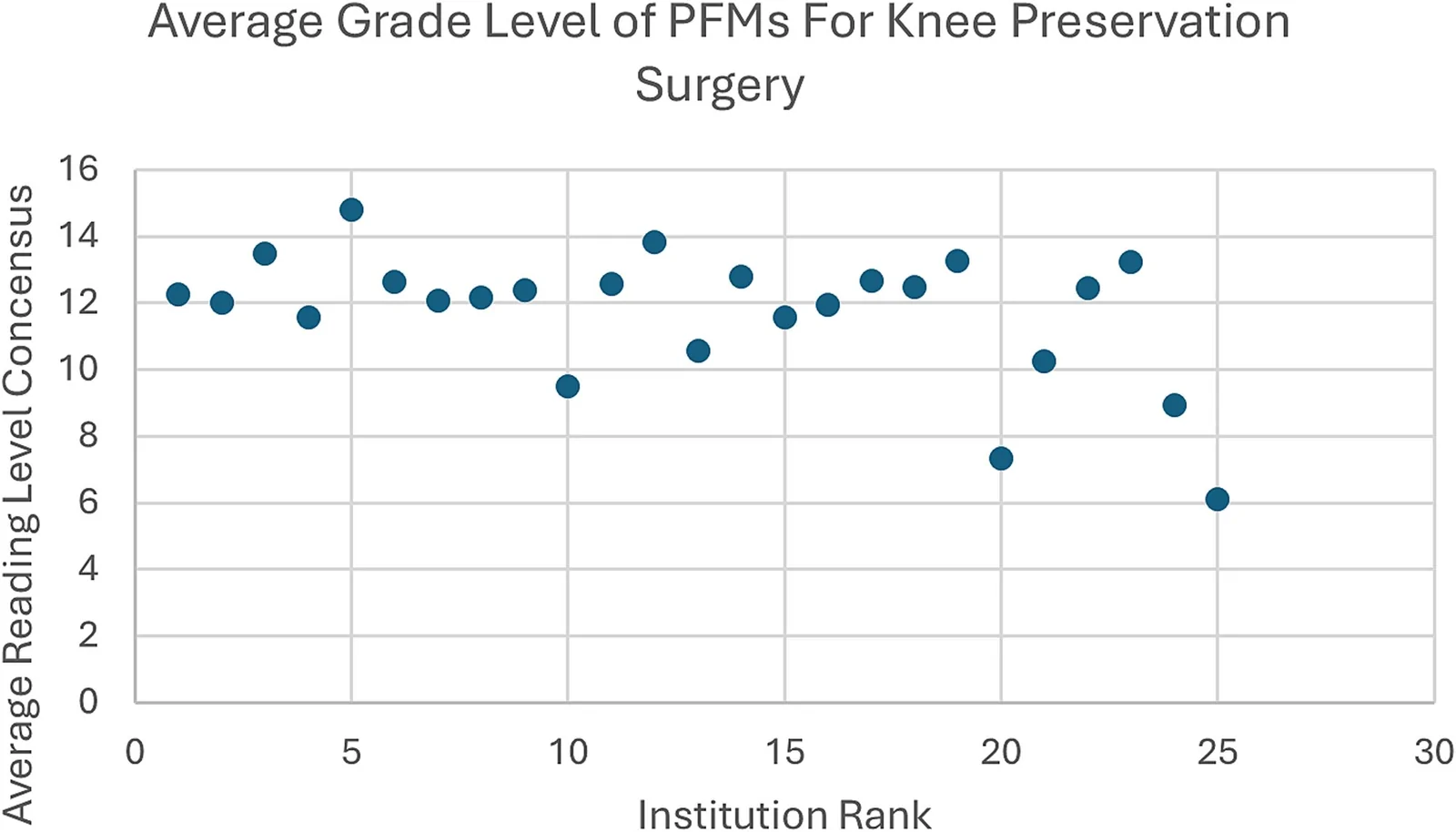
Average grade level of online PFMs for knee preservation surgery provided by top US orthopaedic institutions as graded by the Average Reading Level Consensus. This metric combines 8 validated readability formulas to provide an average reading level of a single text or document.

**Figure 3:**
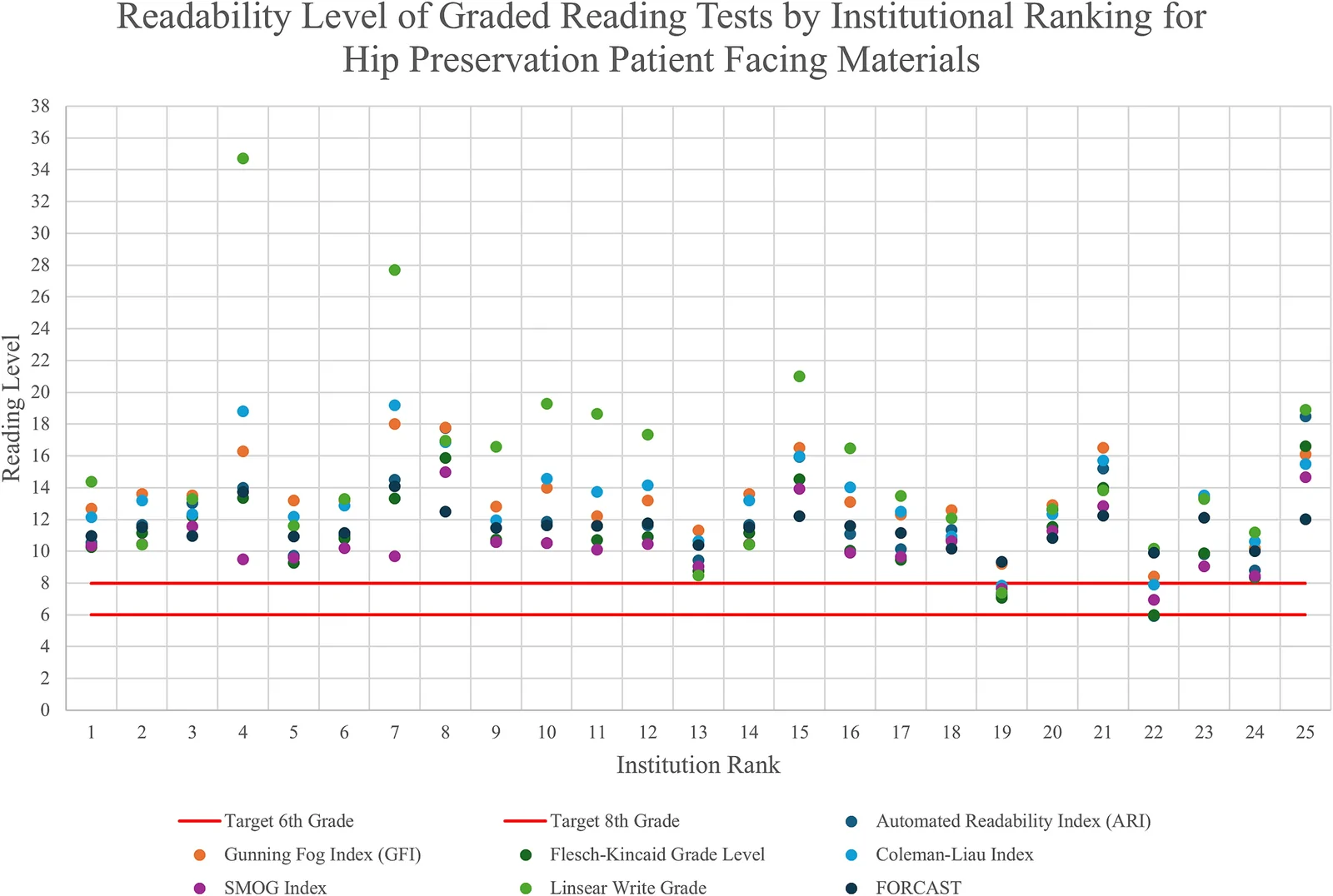
Readability of patient facing materials pertaining to hip preservation published by top 25 orthopaedic institutions with reference lines at 6th and 8th grade target levels.

**Figure 4:**
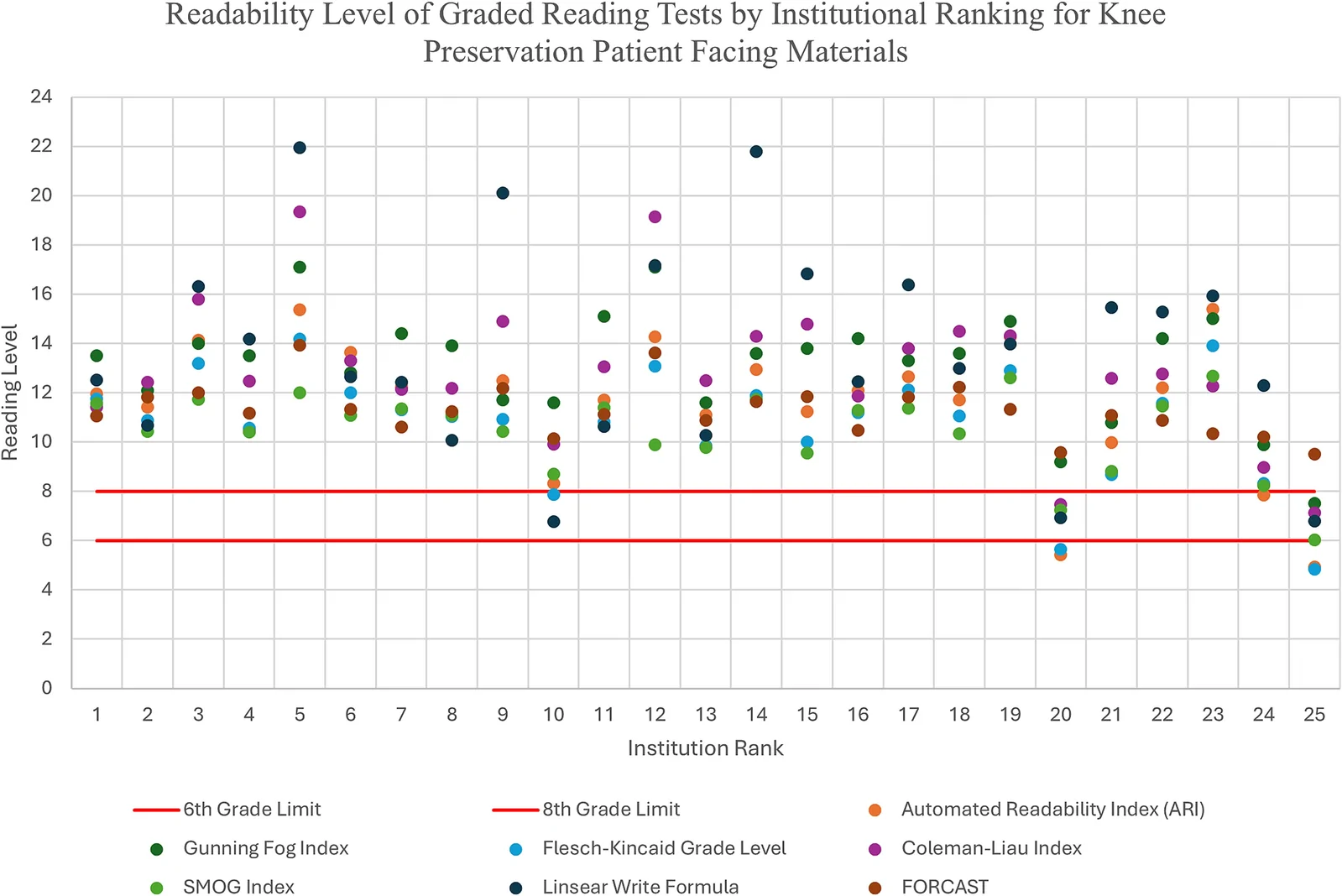
Readability of patient facing materials pertaining to knee preservation published by top 25 orthopaedic institutions with reference lines at 6th and 8th grade target levels.

## DISCUSSION

The topic of joint preservation has gained significant popularity and attention in recent years and the number of procedures pertaining to joint preservation such as arthroscopy, osteotomy, cartilage repair, and restoration has increased dramatically [1]. As such, patient interest in this field is also increasing. For this reason, it is crucial that patients can become well informed about the current state of medicine, their own medical conditions, as well as their treatment options.

PFMs for hip and knee preservation surgery provided by highly ranked orthopaedic institutions were commonly written well above the recommended sixth or eighth grade reading level. Our results confirm that regardless of formula, the content of available PFMs exceed recommended complexity for the general population. This is supported by the collinearity of the data from each readability formula as well as the Average Reading Level Consensus.

The gap between the reading level of available materials and the literacy level of the target patient population may hinder patient comprehension, engagement, and informed consent, potentially impacting healthcare outcomes. To increase accessibility, enhance healthcare literacy, and improve patient outcomes, institutions should simplify these materials to meet recommended readability standards. Analysis of PFMs across a broad range of medical specialties indicates that readability is an almost universal challenge [4, 5, 7, 9, 12]. However, an analysis of online ankle fracture related PFMs demonstrated that such materials were written at approximately an eighth grade reading level, indicating that it is possible for orthopaedic literature to meet the recommended guidelines [14].

This study also identified no significant correlation between institutional ranking and readability for hip preservation material (ρ = −0.10 (*P* = .64)) and knee preservation material (ρ = 0.22 (*P* = .29)). Of note, Parsa *et al*. found that higher institutional ranking was associated with poorer readability, a finding that our study did not support [15]. Furthermore, multivariate analysis did not find any significant correlation between other institutional factors and readability. Our study utilized 10 unique validated readability formulas and all readability metrics were highly collinear (Pearson correlation *r* > 0.8). This analysis indicates that regardless of the evaluation tool, the PFMs evaluated uniformly exceeded recommended grade level.

A particular challenge in the development of online PFMs is that they are typically accessed independently by patients without the opportunity for real time discussion or question and answer. In addition to utilizing various tools to develop readable materials, it is important to address patient needs on an individual basis when they do present to the office. The teach back method is a highly effective way to conduct patient conversations and best ensure comprehension, confidence, and connection between the patient and provider [16]. While this is an inherent challenge to online PFMs, it should serve as a guideline for clinical discussions to best ensure that patients understand their condition, treatment options, and next steps in their care.

This study has multiple relevant limitations. Readability formulas do not completely evaluate all factors relating to patient comprehension. The Average Reading Level Consensus was included in this study to combat any deficiencies in a single formula as this metric utilizes eight of the previous readability formulas. The average grade level accounts for different aspects of readability evaluated by certain specific formulas. Additionally, these formulas have the benefit of serving as a standardized method of evaluation but do not directly evaluate patient comprehension. Direct measurement of patient comprehension could be evaluated in future studies. Finally, this study utilized rankings according to the US News and World Report, which is only one list of top orthopaedic institutions.

This study demonstrates that PFMs for hip and knee preservation surgery are written well above recommended reading levels. General recommendations for the simplification of medical information include substitution of medical terms for simpler plain-language terms, defining medical terms that cannot be substituted, and using multimedia to supplement text information. It is important to note that using plain language is not ‘dumbing down’ material but rather has repeatedly been shown to increase comprehension [2, 8]. Interestingly, AI models such as Chat GPT may also be useful in simplifying PFMs to enhance comprehension and health literacy [11, 17]. In addition to AI models, publicly available tools such as the CDC’s ‘Simply Put’ guidelines can be useful in crafting readable materials [18].

## Data Availability

The data underlying this article are available in the article.
